# Supplementary figures and images for: A genetically engineered mouse model for ovarian hyperstimulation syndrome
Source: PeerJ. 2025 May 26;13:e19531. doi: 10.7717/peerj.19531 (PMC12121618; doi:10.7717/peerj.19531)

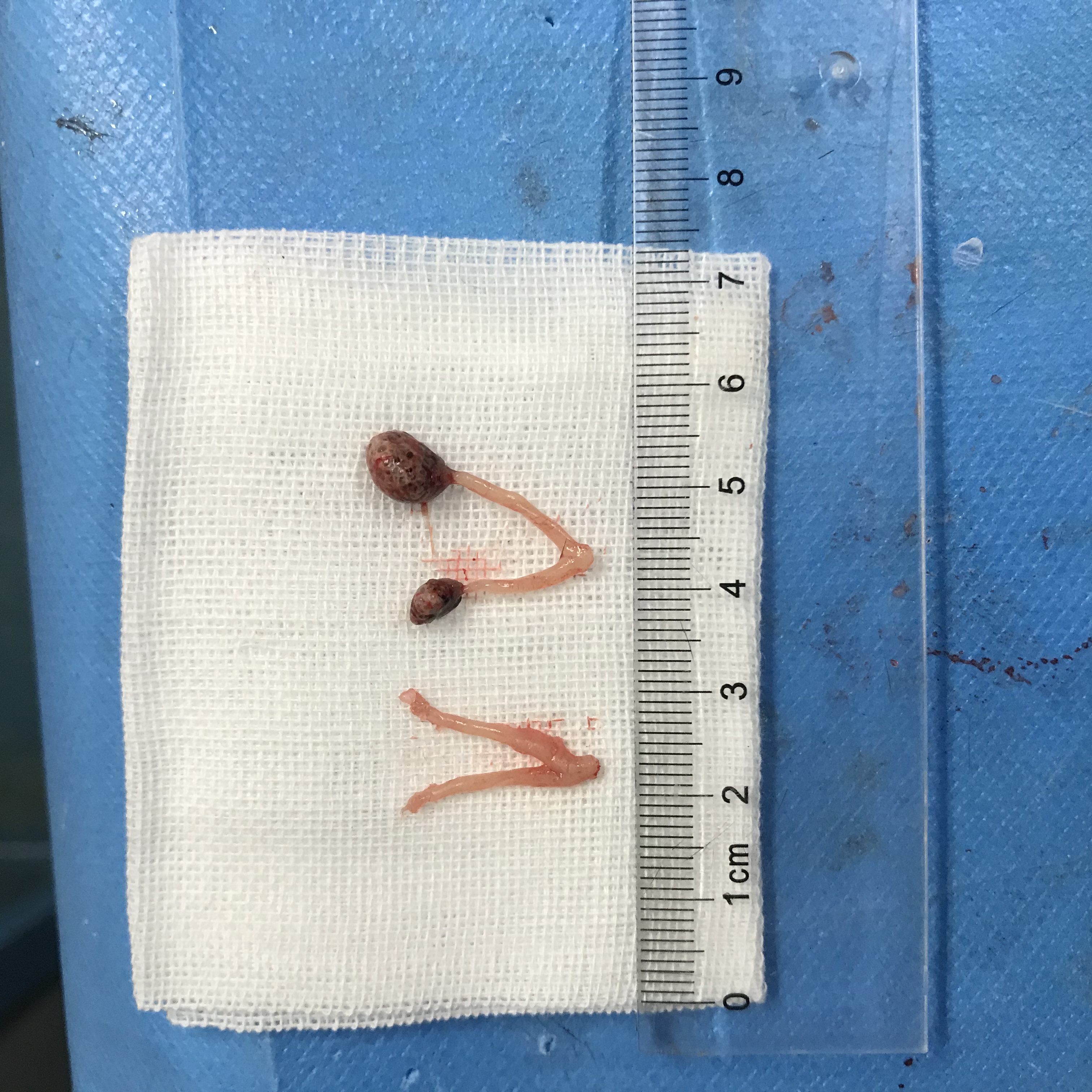

Supplement: Supplemental Information 1 [file peerj-13-19531-s001.zip › rawdata/figure1 b.jpeg]

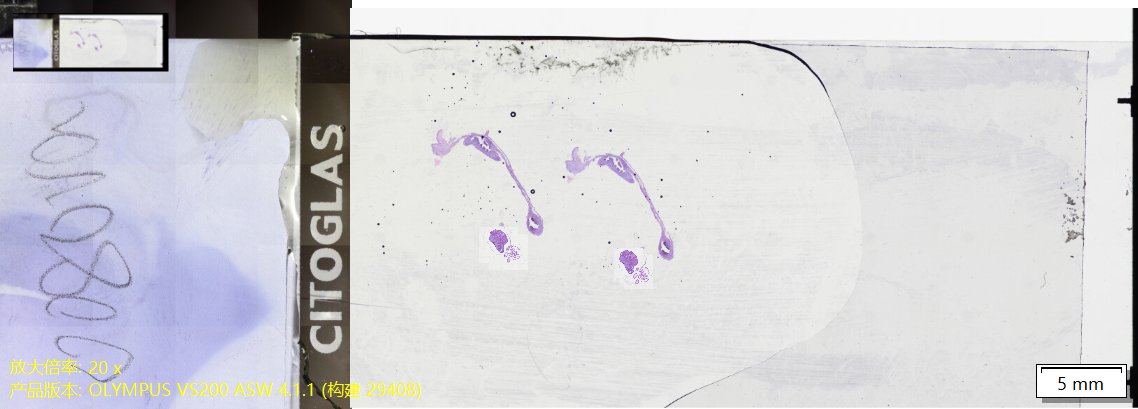

Supplement: Supplemental Information 1 [file peerj-13-19531-s001.zip › rawdata/figure1 d(WT ovary).jpg]

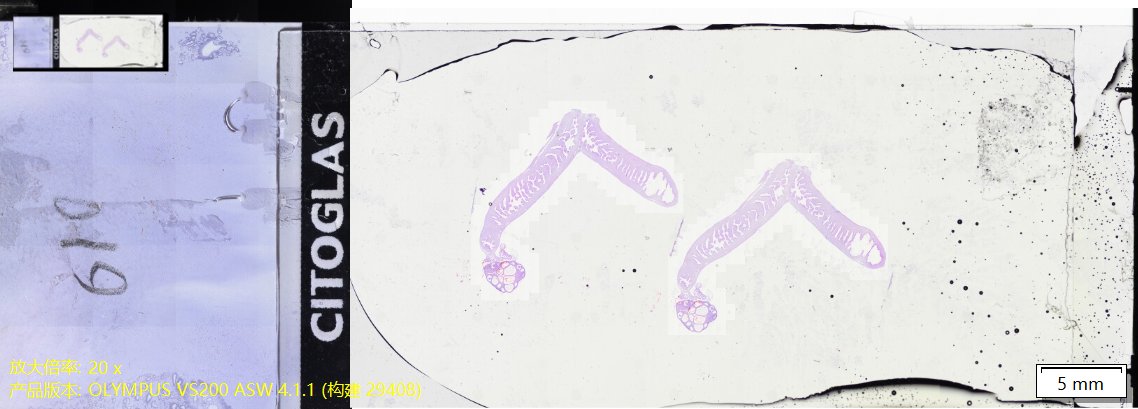

Supplement: Supplemental Information 1 [file peerj-13-19531-s001.zip › rawdata/figure1 e(FKI ovary).jpg]

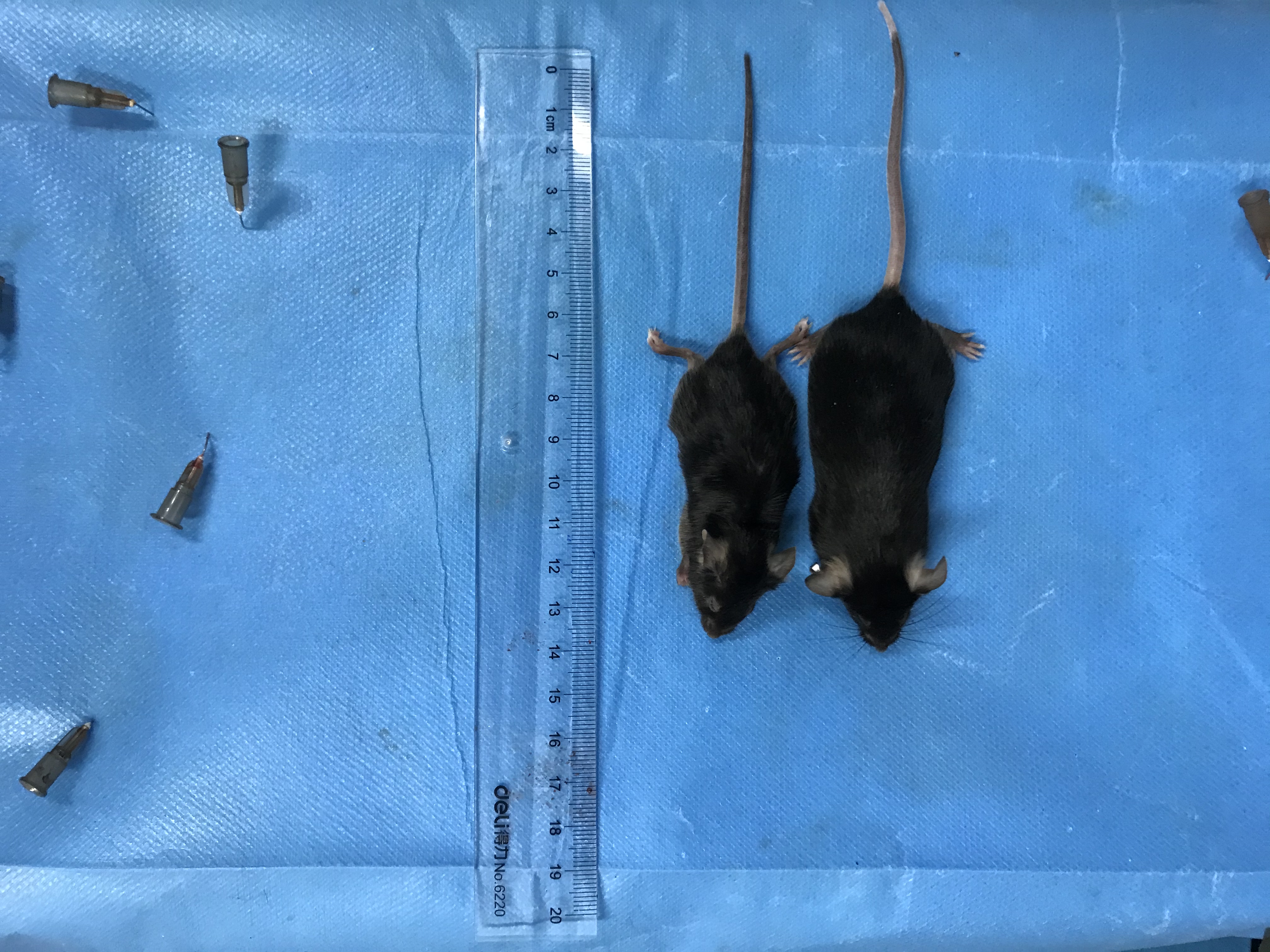

Supplement: Supplemental Information 1 [file peerj-13-19531-s001.zip › rawdata/figure5 a.jpeg]

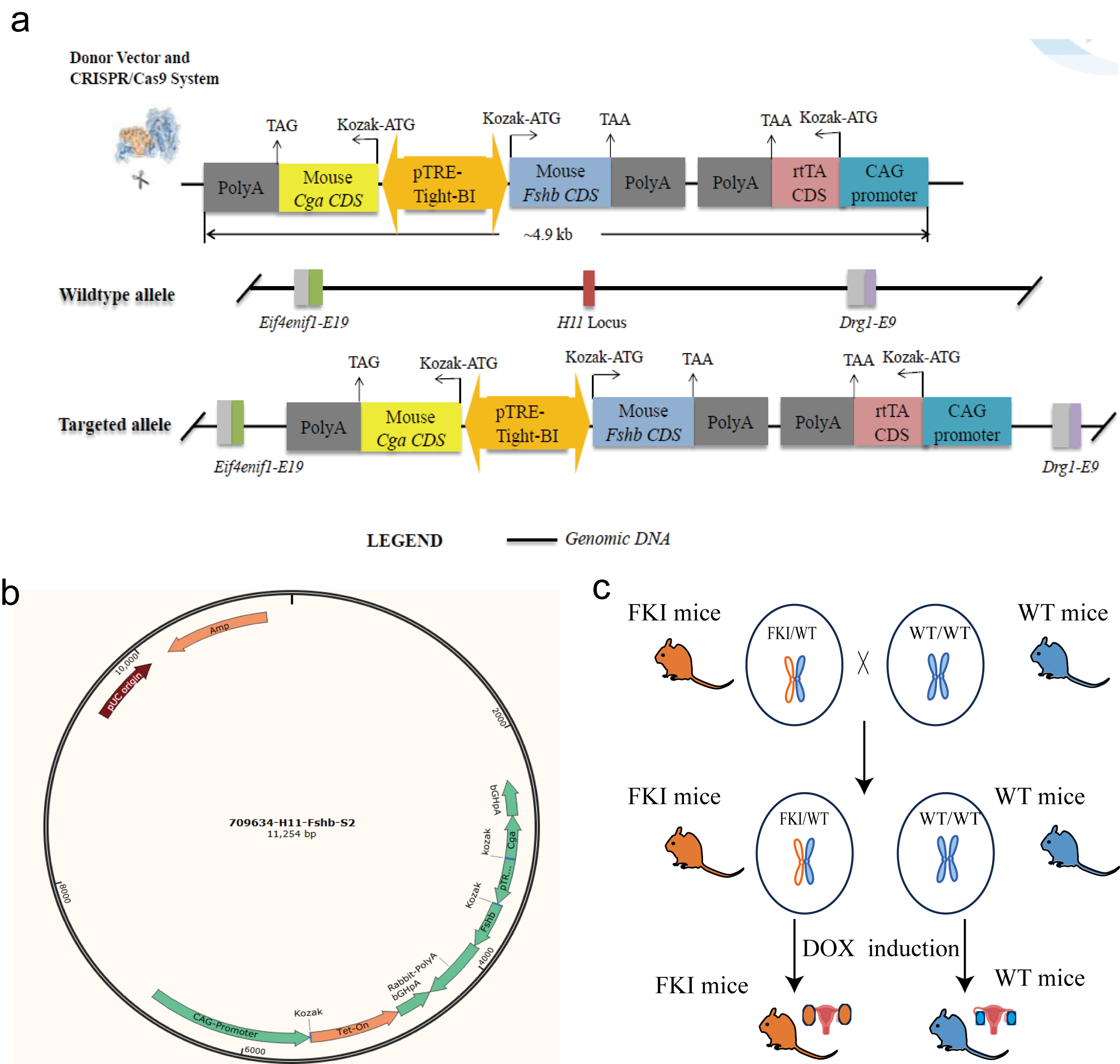

Supplement: Supplemental Information 2 — (a) Schematic design of pTRE-Cga-Fshb-CAG-rtTA fragment. (b) Schematic design of FKI mice model through CRISPR/Cas9 technology. (c) Schematic design of sustaining FKI mice line. [file peerj-13-19531-s002.png]

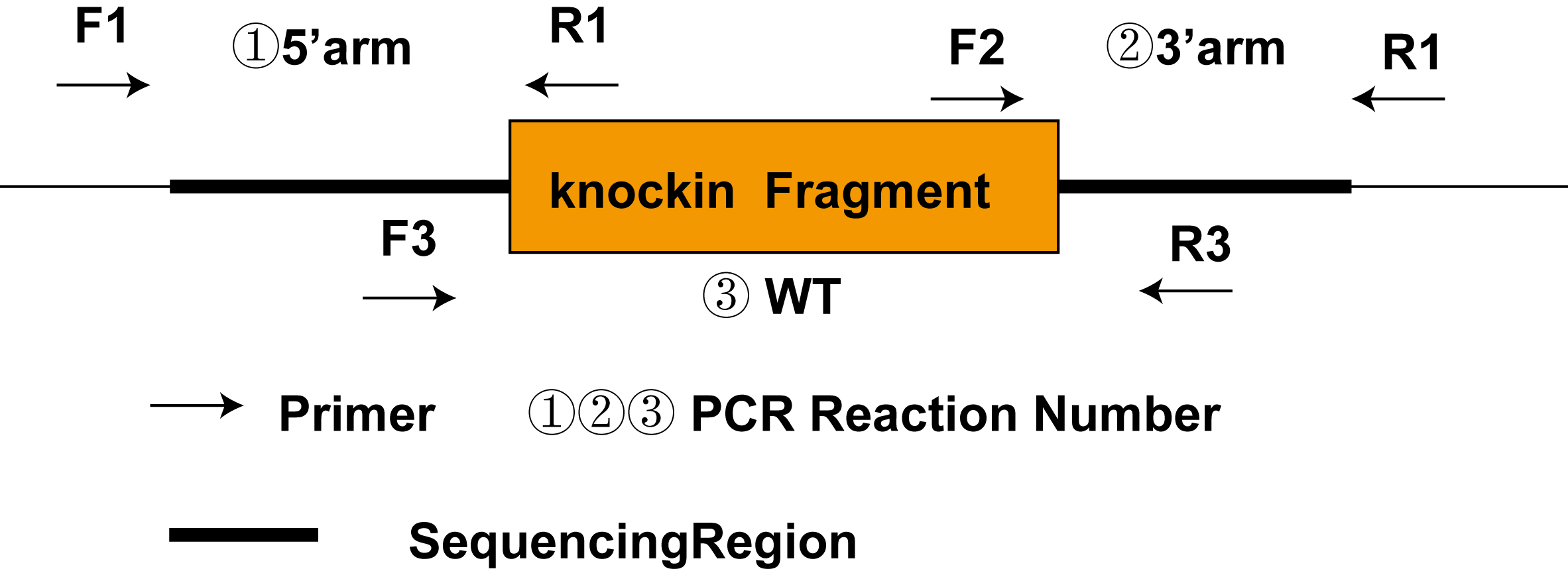

Supplement: Supplemental Information 3 [file peerj-13-19531-s003.png]
