# Supplementary material for: A genetically engineered mouse model for ovarian hyperstimulation syndrome
Source: PeerJ. 2025 May 26;13:e19531. doi: 10.7717/peerj.19531 (PMC12121618; doi:10.7717/peerj.19531)
Supplement: Supplemental Information 4 [file peerj-13-19531-s004.docx]

**Supplement data**

1. **Gene information**
   1. **The serial number of the genes in the database**

Supplemental table1: Database information for genes of interest

| Gene of interest | NCBI number | MGI number |
| --- | --- | --- |
| *Cga* | 12640 | 88390 |
| *Fshb* | 14308 | 95582 |

**1.2 the database link of the genes of interest**

NCBI URL:

https://www.ncbi.nlm.nih.gov/gene/?term=12640

https://www.ncbi.nlm.nih.gov/gene/?term=14308

Ensablebull links:

<http://uswest.ensembl.org/Mus_musculus/Gene/Summary?db=core;g=ENSMUSG00000028298>; r=4:34893779-34907370

http://uswest.ensembl.org/Mus_musculus/Gene/Summary?db=core;g=ENSMUSG00000027120;r=2:107056140-107059656;t=ENSMUST00000028533

**1.3 Transcript information**

Transcript of the program (ENSEMBL number) was CGA-201 (ENSMUST00000029975.9) and FSHB-201 (ENSMUST00000028533.6).

**2.Genetic identification**

Genotypes were identified in all mice by polymerase chain reaction (PCR) amplification of genomic DNA isolated from the tail tip. Mouse genotypes were identified according to the following strategies. Identification was performed by PCR. Primer sequence information was shown in Supplemental Figure. 2 and Supplemental Table2-4.

Supplemental table2: Primer information for genetic identification of mouse

| PCR serial number | Primer number | Primer name | Primer sequences | product |
| --- | --- | --- | --- | --- |
| ①5’arm | F1 | H11-tF2 | ATGCCCACCAAAGTCATCAGTGTAG | WT:0bp  Targeted:1731bp |
|  | R1 | 709634- Cga -5tR2 | CCAAAGCATTTACTAAGGCCACAG |  |
| ②3’arm | F2 | H11- CAG -5tR2 | AGGCGGGCCATTTACCGTAAGTTA | WT:0bp  Targeted: 1294bp |
|  | R2 | H11-tR2 | TCACAGAAACCATATGGCGCTCC |  |
| ③WT | F3 | H11-tF3 | GGGCAGTCTGGTACTTCCAAGCT | WT:285bp  Targeted: 0bp |
|  | R3 | H11-tR3 | ATATCCCCTTGTTCCCTTTCTGC |  |

Supplemental table3: PCR reaction component

| Seg. | reaction component | Volume (µl) |
| --- | --- | --- |
| 1 | 2×Taq Master Mix, Dye Plus, (Vazyme P112-03) | 12.5 |
| 2 | ddH2O | 9.5 |
| 3 | Primer A(10pmol/µl) | 1 |
| 4 | Primer B(10pmol/µl) | 1 |
| 5 | Template(≈100ng/μl) | 1 |

Supplemental table4: PCR program

| Seg. | Temp. | Time | Cycle |
| --- | --- | --- | --- |
| 1 | 95℃ | 5min |  |
| 2 | 98℃ | 30s | 20× |
| 3 | 65℃（-0.5℃/cycle） | 30s |  |
| 4 | 72℃ | 45s |  |
| 5 | 98℃ | 30s | 20× |
| 6 | 55℃ | 30s |  |
| 7 | 72℃ | 45s |  |
| 8 | 72℃ | 5min |  |
| 9 | 10℃ | hold |  |
